# High-performance silicon photonic tri-state switch based on balanced nested Mach-Zehnder interferometer

**DOI:** 10.1038/s41598-017-12455-8

**Published:** 2017-09-25

**Authors:** Zeqin Lu, Dritan Celo, Hamid Mehrvar, Eric Bernier, Lukas Chrostowski

**Affiliations:** 1 Department of Electrical and Computer Engineering, University of British Columbia (UBC), Vancouver, Canada; 2 Central Research Institute, Huawei Technologies Canada Co., Ltd, Ottawa, Canada

## Abstract

This work proposes a novel silicon photonic tri-state (cross/bar/blocking) switch, featuring high-speed switching, broadband operation, and crosstalk-free performance. The switch is designed based on a 2 × 2 balanced nested Mach-Zehnder interferometer structure with carrier injection phase tuning. As compared to silicon photonic dual-state (cross/bar) switches based on Mach-Zehnder interferometers with carrier injection phase tuning, the proposed switch not only has better performance in cross/bar switching but also provides an extra blocking state. The unique blocking state has a great advantage in applications of *N* × *N* switch fabrics, where idle switching elements in the fabrics can be configured to the blocking state for crosstalk suppression. According to our numerical experiments on a fully loaded 8 × 8 dilated Banyan switch fabric, the worst output crosstalk of the 8 × 8 switch can be dramatically suppressed by more than 50 dB, by assigning the blocking state to idle switching elements in the fabric. The results of this work can extend the functionality of silicon photonic switches and significantly improve the performance of on-chip *N* × *N* photonic switching technologies.

## Introduction

Global data traffic has been exploding in recent years, putting increased pressure on data centre communication systems. To meet the rapid growth of data traffic, data switching technologies require larger switching capacity and higher interconnect bandwidth. Silicon-on-insulator (SOI) is a low-cost platform to develop high-performance optical switching technologies for data centre communication applications. For such applications, switches that are high-speed, broadband, and have low crosstalk are highly desired.

**Figure 1:**
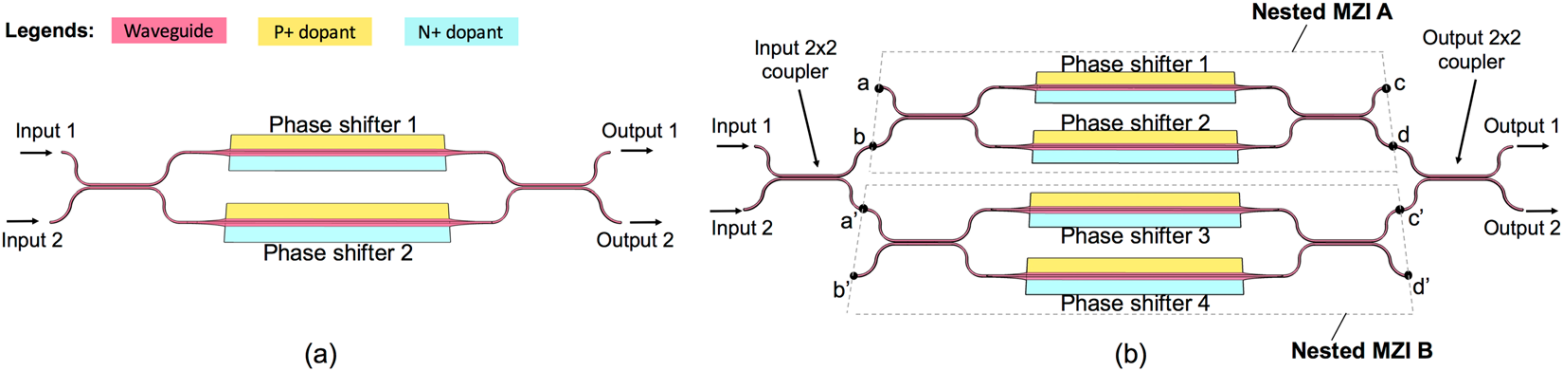
(**a**) Schematic for a Mach-Zehnder interferometer (MZI) switch; (**b**) Schematic for the proposed balanced nested Mach-Zehnder interferometer (BNMZI) switch.

On SOI platforms, Mach-Zehnder interferometers (MZIs) are widely used for broadband switch design. The schematic for such a device is shown in Fig. 1a. To realize high-speed switching, the phase shifter of a MZI switch is typically designed using a P-i-N diode operating in a carrier injection mode^1–6^. While providing an efficient optical phase shift with a speed of hundreds of MHz, carrier injection also produces inherent insertion loss due to free-carrier absorption^7^. This is problematic for a MZI switch operating at the bar state, where one of its phase shifters is tuned by a *π* phase shift while the other is not. In such a state, the optical power in the two interferometer arms of the switch is unbalanced, leading to a large crosstalk at the cross output port^1–6^. In order to suppress crosstalk, the optical power inside a MZI switch needs to be balanced. Recently, a solution^8^ using a nested MZI structure with a variable optical attenuator was proposed. Although this solution balances the optical power in the switch and therefore achieves low crosstalk performance, the optical bandwidth of the switch is strongly limited by its asymmetric interferometer structure. To the best of our knowledge, there is no known efficient method to balance the optical power of a carrier injection based MZI switch to achieve low crosstalk performance while maintaining its optical bandwidth. In this work, we propose a carrier injection based SOI switch, which not only has broadband and crosstalk-free performance but also provides three different switching states: cross, bar, and blocking.

## Operation principle of proposed BNMZI switch

Figure 1b shows the schematic for the proposed switch, which is a 2 × 2 device based on a balanced nested Mach-Zehnder interferometer (BNMZI) structure. Such a device consists of an input 2 × 2 coupler, an output 2 × 2 coupler, and two balanced main interference arms with each being a balanced 2 × 2 MZI, i.e., nested MZI A and nested MZI B as shown in Fig. 1b. Each nested MZI has two identical carrier injection phase shifters. The BNMZI switch has a balanced architecture, in which the optical path lengths are equal in its two main interference arms; as a result, the optical operation bandwidth is not limited by the switch architecture, i.e., broadband performance can be achieved.

The transfer matrix method is used to analyze the operation principle of the proposed BNMZI switch. The relationship between the input and output electric fields of the nested MZIs can be expressed as:

1 $$[\begin{array}{c}{E}_{c}\\ {E}_{d}\end{array}]=[\begin{array}{cc}t & -j\kappa \\ -j\kappa  & t\end{array}]\,[\begin{array}{cc}{\alpha }_{1}{e}^{-j{\varphi }_{1}} & 0\\ 0 & {\alpha }_{2}{e}^{-j{\varphi }_{2}}\end{array}]\,[\begin{array}{cc}t & -j\kappa \\ -j\kappa  & t\end{array}]\,[\begin{array}{c}{E}_{a}\\ {E}_{b}\end{array}],$$

2 $$[\begin{array}{c}{E}_{{c}^{{\rm{^{\prime} }}}}\\ {E}_{{d}^{{\rm{^{\prime} }}}}\end{array}]=[\begin{array}{cc}t & -j\kappa \\ -j\kappa  & t\end{array}]\,[\begin{array}{cc}{\alpha }_{3}{e}^{-j{\varphi }_{3}} & 0\\ 0 & {\alpha }_{4}{e}^{-j{\varphi }_{4}}\end{array}]\,[\begin{array}{cc}t & -j\kappa \\ -j\kappa  & t\end{array}]\,[\begin{array}{c}{E}_{{a}^{{\rm{^{\prime} }}}}\\ {E}_{{b}^{{\rm{^{\prime} }}}}\end{array}],$$

where *E*_*x *=* a*,*b*,*c*,*d*..._ is the electric field at port *x* of the nested MZIs, as illustrated in Fig. 1b; *t* and *κ* are through-coupling coefficient and cross-coupling coefficient for each 2 × 2 coupler, respectively, and we assume that all the 2 × 2 couplers in the BNMZI switch are identical and are lossless, i.e., *t* ^2^ + *κ* ^2^ = 1; *ϕ*_*i* = 1,2,3,4_ is the modulated optical phase shift of phase shifter *i*; *α*_*i* = 1,2,3,4_ is optical field transmission factor of phase shifter *i*, and it represents the optical attenuation due to free-carrier absorption. In our design as shown in Fig. 1b, ports a and b′ are both terminated, i.e., *E*_*a*_ = *E*_*b*′_ = 0, and accordingly we obtain the electric field transfer functions for the light paths from ports b to d and from ports a′ to c′, which are:

3 $$\frac{{E}_{d}}{{E}_{b}}=-{\kappa }^{2}{\alpha }_{1}{e}^{-j{\varphi }_{1}}+{t}^{2}{\alpha }_{2}{e}^{-j{\varphi }_{2}},$$

4 $$\frac{{E}_{{c}^{{\rm{^{\prime} }}}}}{{E}_{{a}^{{\rm{^{\prime} }}}}}=-{\kappa }^{2}{\alpha }_{4}{e}^{-j{\varphi }_{4}}+{t}^{2}{\alpha }_{3}{e}^{-j{\varphi }_{3}}.$$

The relationship between the input and output electric fields of the BNMZI switch can be given by:

5 $$[\begin{array}{c}{E}_{out1}\\ {E}_{out2}\end{array}]=[\begin{array}{cc}t & -j\kappa \\ -j\kappa  & t\end{array}]\,[\begin{array}{cc}\frac{{E}_{d}}{{E}_{b}} & 0\\ 0 & \frac{{E}_{c^{\prime} }}{{E}_{a^{\prime} }}\end{array}]\,[\begin{array}{cc}t & -j\kappa \\ -j\kappa  & t\end{array}]\,[\begin{array}{c}{E}_{in1}\\ {E}_{in2}\end{array}].$$

Assuming light is launched into input 1 of the switch, i.e., *E*_*in*1_ = 1 and *E*_*in*2_ = 0, the power at the two outputs are given by:

6 $$|{E}_{out1}{|}^{2}=|-{t}^{2}{\kappa }^{2}{\alpha }_{1}{e}^{-j{\varphi }_{1}}+{t}^{4}{\alpha }_{2}{e}^{-j{\varphi }_{2}}-{t}^{2}{\kappa }^{2}{\alpha }_{3}{e}^{-j{\varphi }_{3}}+{\kappa }^{4}{\alpha }_{4}{e}^{-j{\varphi }_{4}}{|}^{2},$$

7 $$|{E}_{out2}{|}^{2}=|-j\kappa t(-{\kappa }^{2}{\alpha }_{1}{e}^{-j{\varphi }_{1}}+{t}^{2}{\alpha }_{2}{e}^{-j{\varphi }_{2}}+{t}^{2}{\alpha }_{3}{e}^{-j{\varphi }_{3}}-{\kappa }^{2}{\alpha }_{4}{e}^{-j{\varphi }_{4}}{)|}^{2}.$$

The nested MZIs A and B of the BNMZI switch can be driven in a balanced manner in order to balance the free-carrier absorption induced insertion loss in the switch, and therefore, the switch can be crosstalk-free. Throughout this paper, we define crosstalk as the transmission power at the unintended switch output. The operation principle of the BNMZI switch is described as follows:

### Cross state

**Figure 2:**
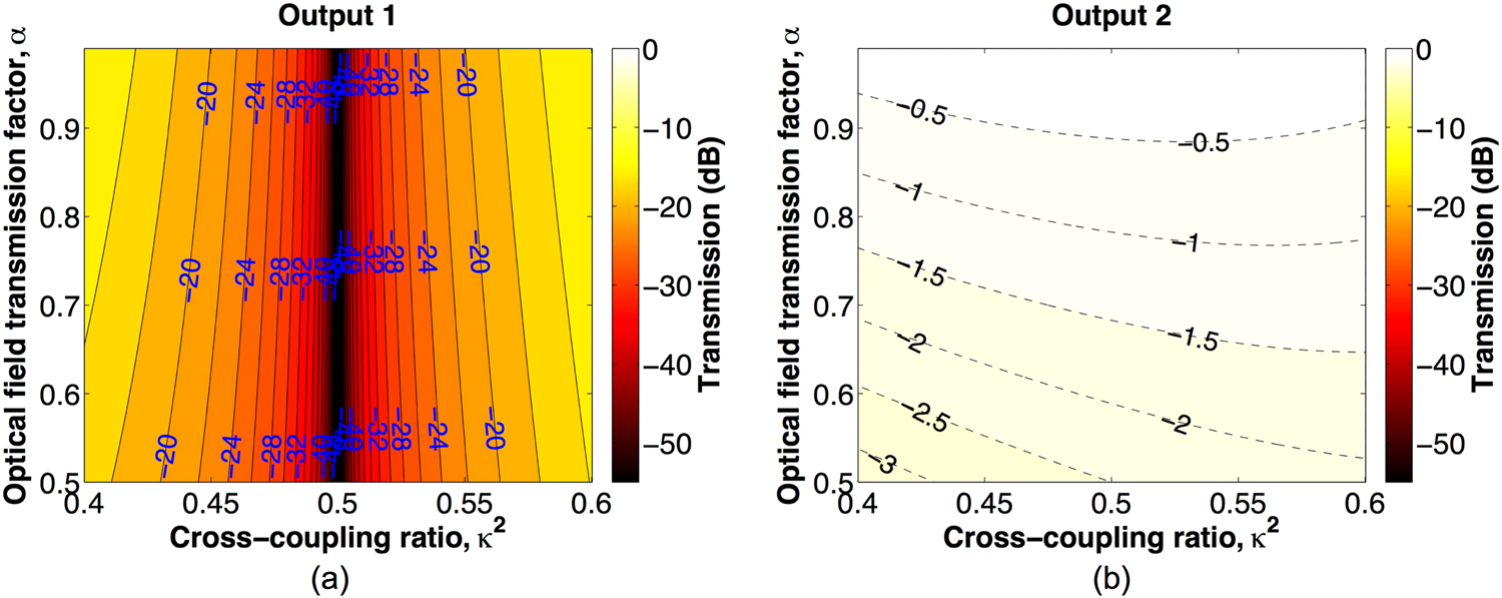
Cross state output transmissions as a function of *κ* ^2^ and *α*. (**a**) Output 1. (**b**) Output 2.

When *ϕ*_1_ = *ϕ*_4_ = 0 and *ϕ*_2_ = *ϕ*_3_ = *π*, we have *α*_1_ = *α*_4_ = 1 and *α*_2_ = *α*_3_ = *α*, where *α* is optical field transmission factor for the *π* phase tuning and is dependent on the design parameters of the phase shifter, e.g., phase shifter length and optical confinement of the waveguide. In such phase tuning, the nested MZI A routes light from ports b to d (see Fig. 1b) with a digital *π* phase shift, and the nested MZI B routes light from ports a′ to c′ (see Fig. 1b) also with a digital *π* phase shift. As a result, the BNMZI switch operates in the cross state^9^. In addition, the insertion loss of the two nested MZIs are balanced in such balanced phase tuning so that the BNMZI switch can be crosstalk-free. Based on Eqs (6) and (7), we calculate the optical output transmissions as a function of *κ* ^2^ and *α*, and the results for output 1 and output 2 are shown in Fig. 2a and b, respectively. According to the results, light launched at input 1 is cross-switched to output 2. Ideally, when the 2  × 2 couplers of the BNMZI switch are perfect 3-dB couplers, i.e., *κ* ^2^ = *t* ^2^ = 0.5, the output transmissions are given by:

8 $$|{E}_{out1}{|}^{2}\,=\,\mathrm{0;}\,\,\,\,|{E}_{out2}{|}^{2}=\frac{1}{4}{\mathrm{(1}+\alpha )}^{2},$$

which show crosstalk-free performance for any *α*.

### Bar state

**Figure 3:**
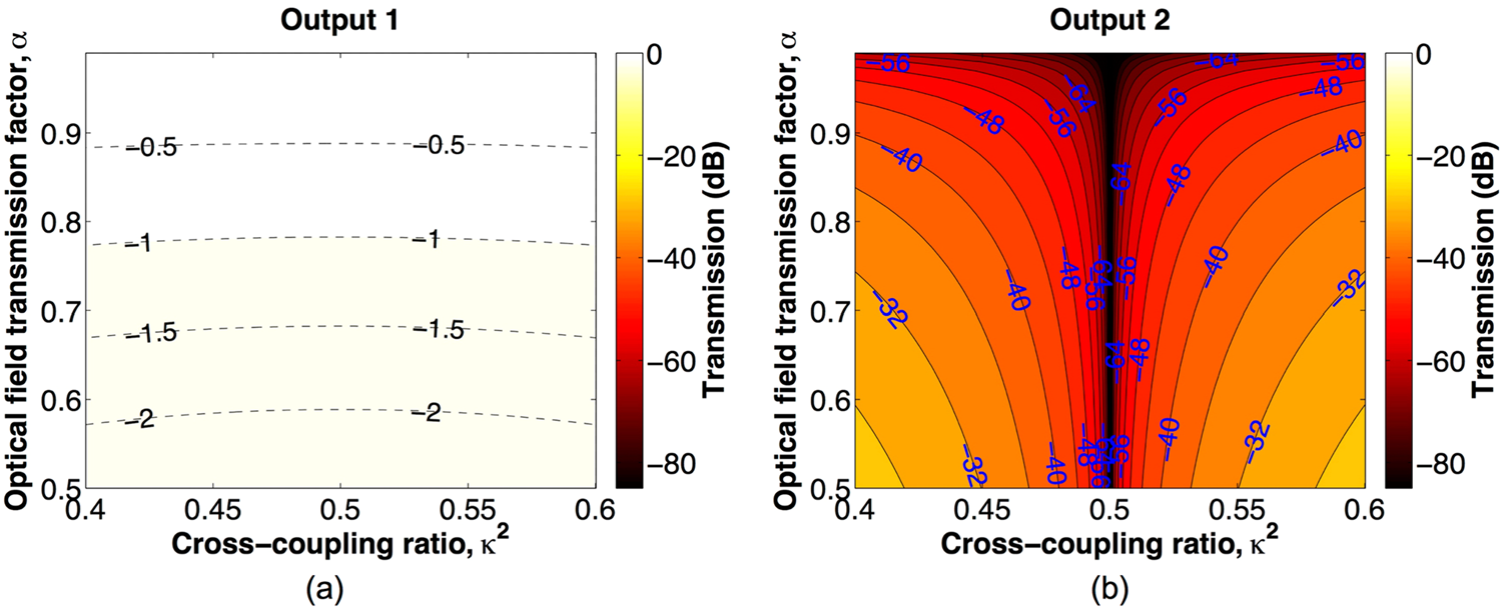
Bar state output transmissions as a function of *κ* ^2^ and *α*. (**a**) Output 1. (**b**) Output 2.

When *ϕ*_1_ = *ϕ*_3_ = *π* and *ϕ*_2_ = *ϕ*_4_ = 0, we have *α*_1_ = *α*_3_ = *α* and *α*_2_ = *α*_4_ = 1. In such phase tuning, the nested MZI A routes light from ports b to d (see Fig. 1b) with a digital 0 phase shift, while the nested MZI B routes light from ports a′ to c′ (see Fig. 1b) with a digital *π* phase shift. As a result, the BNMZI switch operates in the bar state. And more, the insertion loss of the two nested MZIs are balanced in such balanced phase tuning so that the BNMZI switch can be crosstalk-free. Based on Eqs (6) and (7), we calculate the optical output transmissions as a function of *κ* ^2^ and *α*, and the results for output 1 and output 2 are shown in Fig. 3a and b, respectively. As can be seen from the results, light launched at input 1 is routed to output 1. Ideally, when *κ* ^2^ = *t* ^2^ = 0.5, the output transmissions are given by:

9 $$|{E}_{out1}{|}^{2}=\frac{1}{4}{\mathrm{(1}+\alpha )}^{2};\,\,\,|{E}_{out2}{|}^{2}\,=\,0.$$

In this case, the switch is crosstalk-free for any *α*.

### Blocking state

**Figure 4:**
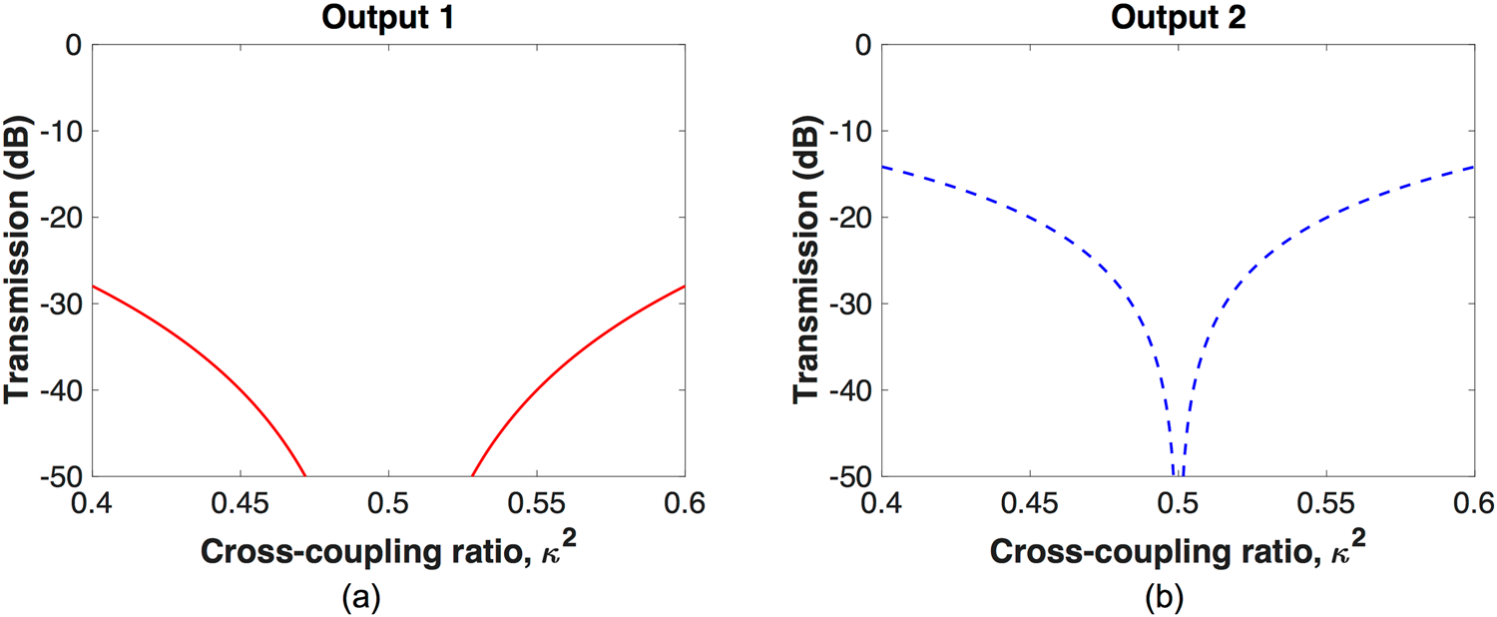
Blocking state output transmissions as a function of *κ* ^2^. (**a**) Output 1. (**b**) Output 2.

When no phase tuning is applied, i.e., *ϕ*_1_ = *ϕ*_2_ = *ϕ*_3_ = *ϕ*_4_ = 0, we have ${\alpha }_{1}={\alpha }_{2}={\alpha }_{3}={\alpha }_{4}\,=\,1$. In such a state, the nested MZI A routes light from ports b to c (see Fig. 1b) and the nested MZI B routes light from ports a′ to d′ (see Fig. 1b), as each nested MZI operates in a cross state. No light is routed to the switch outputs. Both the ports c and d′ can be terminated using waveguide terminators. Based on Eqs (6) and (7), we calculate the optical transmissions at the outputs as a function of *κ* ^2^, and the results for output 1 and output 2 are shown in Fig. 4a and b, respectively. According to the results, the optical transmissions at the two outputs are both low, indicating that the input light is blocked from reaching the two outputs. Ideally, when *κ* ^2^ = *t* ^2^ = 0.5, we obtain:

10 $$|{E}_{out1}{|}^{2}=|{E}_{out2}{|}^{2}\,=\,\mathrm{0,}$$

which shows that the switch is completely blocked.

Table 1 summarizes the phase tunings for the three switching states. Note that in all of the three switching states, the deviation of *κ* ^2^ (*κ* ^2^ ≠ 0.5) breaks the balance of power in the switch and consequently causes switching crosstalk, which are shown in Figs 2a, 3b, 4a and 4b. However, this is an issue due to the imperfect performance of the 2 × 2 3-dB couplers rather than a performance limitation of the proposed BNMZI switch.

**Table 1:** Phase tuning at the switching states of BNMZI switch.

Switching state	*ϕ*_1_	*ϕ*_2_	*ϕ*_3_	*ϕ*_4_
Cross	0	*π*	*π*	0
Bar	*π*	0	*π*	0
Blocking	0	0	0	0

## Results and Discussion

**Figure 5:**
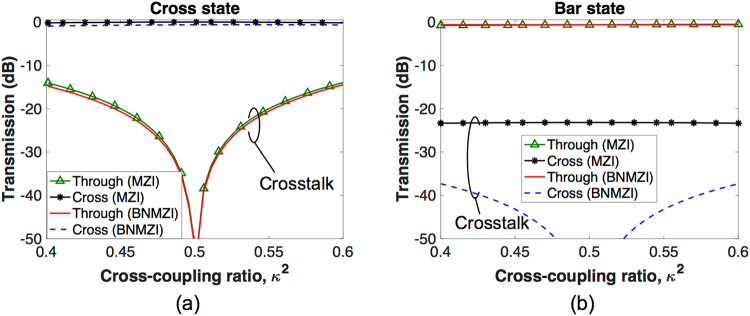
Performance comparison for the MZI switch and the BNMZI switch at the (**a**) cross state and (**b**) bar state.

We compare the proposed BNMZI switch shown in Fig. 1b with the MZI switch shown in Fig. 1a by investigating the impacts of *κ* ^2^ variations on their cross/bar switching performance. On SOI platforms, carrier injection phase shifters^1–6^ have similar insertion losses for a *π* phase shift. In the comparisons, each phase shifter in the two switches is assumed to be 250 *μ*m long with an optical field confinement factor of 0.7, being similar to a demonstrated design^4^. According to Soref’s equations^7,10^, such a phase shifter design requires a carrier density change of 1.162 × 10^18^ (for both electrons and holes) for a *π* phase shift in optical C-band, and correspondingly its free-carrier absorption induced insertion loss translates into *α* = 0.8613. Ideally, we assume that the 2 × 2 couplers in each switch have identical coupling strength due to a uniform fabrication variation across the switch. Figure 5a compares the cross state performance of the two switches, and it is found that the BNMZI switch exhibits slightly lower crosstalk than the MZI switch. Figure 5b compares the bar state performance of the two switches. According to the results, the crosstalk of the MZI switch is greater than −23 dB for a *κ* ^2^ range from 0.4 to 0.6. Meanwhile the crosstalk of the BNMZI switch is well below −37 dB in the same *κ* ^2^ range, being much lower than that of the MZI switch. For a BNMZI switch design using high-performance 3-dB couplers^11^ that have *κ* ^2^ in between 0.48 to 0.52, the crosstalk can be reduced to below −50 dB. According to the comparisons in above, the proposed BNMZI switch exhibits better performance than the MZI switch. In practice, the 2 × 2 couplers of each switch might have different coupling strengths due to random fabrication errors. In such case, the performance of the BNMZI switch and the MZI switch can be analyzed using Monte Carlo (MC) simulations (see Supplementary Information).

In the MZI switch, the insertion loss at the cross state is less than that at the bar state, which is shown in Fig. 5a and b, due to the fact that the driving of MZI switch is imbalanced (the bar state requires driving but the cross state does not). According to Fig. 5a and b, the proposed BNMZI switch has balanced insertion loss at the cross state and the bar state due to its balanced driving scheme. The balanced insertion loss of our BNMZI switch could be beneficial, as it has less variation in loss while changing switching states. For example, in a *N* × *N* switch fabric, different connection paths pass through different numbers of cross state switches and bar state switches, but they can have similar and consistent insertion losses.

**Figure 6:**
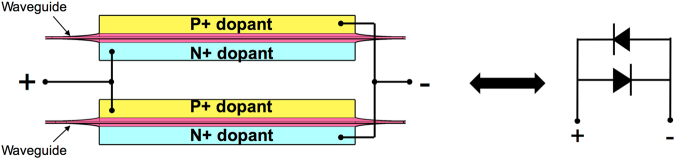
A push-pull driving configuration for each nested MZI.

It should be noted that the proposed BNMZI switch has four phase shifters, and therefore it is less compact in footprint and more complex in electrical control compared to the MZI switch that has only two phase shifters. One method to simplify the electrical controls of our BNMZI switch is by configuring the two carrier injection phase shifters of each nested MZI into a push-pull structure, as illustrated in Fig. 6, which can reduce the electrical controls by half. At the cross state and the bar state, the electrical driving to each push-pull structure turns on one of the P-i-N diodes at a time to provide a *π* phase shift. At the blocking state, there is no driving to the push-pull structures. In practice, the switch phase arms may have initial phase errors due to fabrication variations. In such case, thermo-optic phase correction would be required for each phase arm.

As presented in Eqs (8), (9), and (10), ideally the BNMZI switch can be crosstalk-free if its 2 × 2 couplers have perfect 3-dB coupling ratios. However, most SOI 2 × 2 couplers exhibit unbalanced coupling ratios due to either imperfect designs or fabrication variability, and as a result, crosstalk may exist for a BNMZI switch in practice. According to the results of theoretical analysis shown in Figs 2, 3 and 4, Table 2 presents the performance of an example BNMZI switch having *α* = 0.8613 for a *π* phase tuning and *κ* ^2^ = 0.48 for its 2 × 2 couplers (based on the coupling ratios of a demonstrated 3-dB coupler^11^ in the optical C-band). For a *N* × *N* photonic switch fabric built on imperfect switching elements, crosstalk exists when all signals entering the fabric have the same wavelength, and crosstalk can be accumulated along a connection route from an input to an output, which degrades the performance of the *N* × *N* switch. The unique blocking state of the BNMZI switch has potential in crosstalk suppression for *N* × *N* switch fabrics, such as dilated Banyan^12,13^, hybrid dilated Benes^14^, and route-and-select^15^ architectures. The amount of crosstalk suppression depends on both the switching architecture and the connection loading of the switch fabric. For instance, a fully loaded dilated Banyan architecture has a total of 2 *N*(*N* − 1) switching elements but only uses 2*N*·*log*_2_ *N* switching elements for connections. Idle switches in the fabric can be assigned to the blocking state for crosstalk suppression. For dilated Benes and hybrid dilated Benes architectures, idle switches exist in partially loaded switch fabric. The first order crosstalk in these architectures can be eliminated by constraining connection routing to one signal per switching element^14^; the second and higher order crosstalk can be suppressed by assigning blocking state to the idle switching elements.

**Table 2:** Performance of an example BNMZI switch with *κ* ^2^ = 0.48 and *α* = 0.8613.

	Cross state	Bar state	Blocking state
Through-port transmission (dB)	−28.61	−0.62	−55.92
Cross-port transmission (dB)	−0.65	−51.14	−27.97

**Figure 7:**
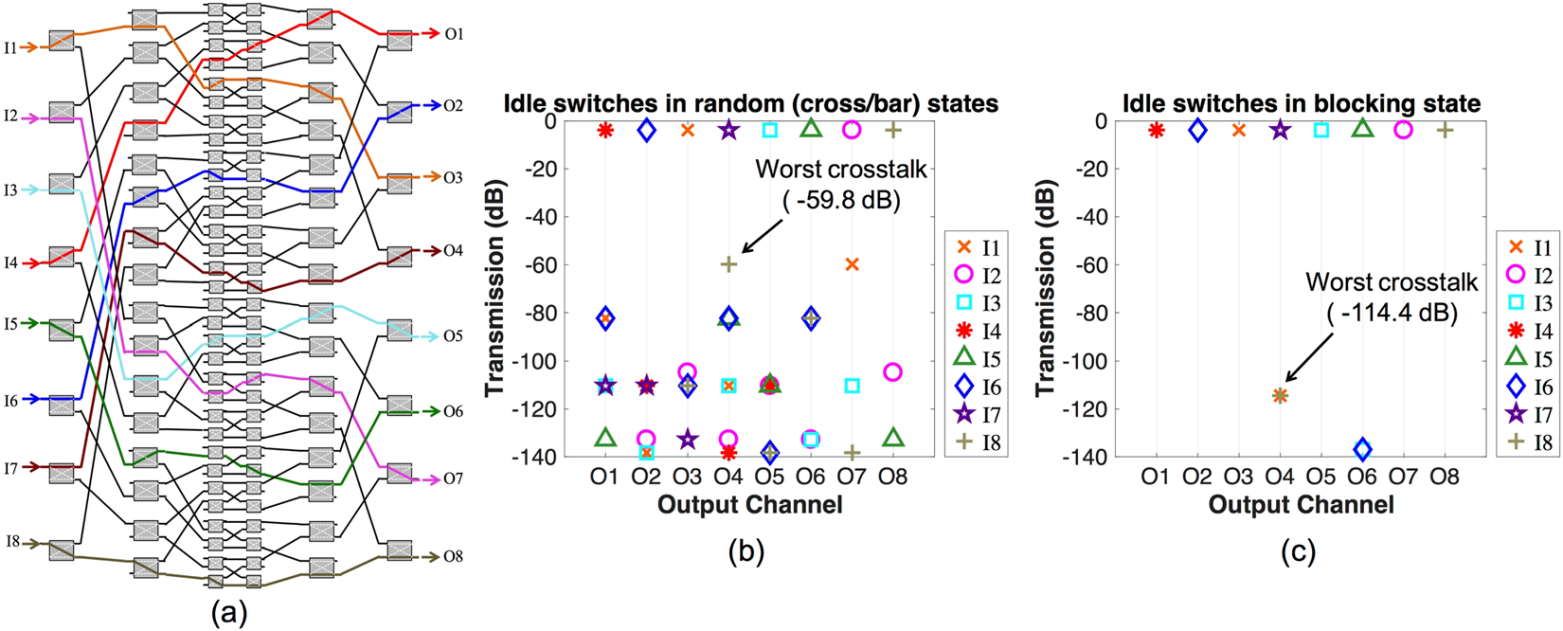
(**a**) Schematic of an example 8 × 8 dilated Banyan switch fabric with established connections: I1-O3, I2-O7, I3-O5, I4-O1, I5-O6, I6-O2, I7-O4, and I8-O8. (**b**) Output transmissions of the 8 × 8 switch without blocking the idle switches (idle switches are randomly in the cross or bar states). (**c**) Output transmissions of the 8 × 8 switch with idle switches being blocked.

As an example, we simulate the performance of a fully loaded 8 × 8 dilated Banyan switch, which uses the 2 × 2 BNMZI switch presented in Table 2 as its switching elements, and we compare its switching crosstalk in the cases with and without assigning blocking states to the idle switches. Figure 7a illustrates the schematic of the 8 × 8 dilated Banyan switch with established connections: I1-O3, I2-O7, I3-O5, I4-O1, I5-O6, I6-O2, I7-O4, and I8-O8, where idle switches can be seen in the connections. Figure 7b shows the simulated output transmissions of the 8 × 8 switch where the idle switches are randomly in the cross or bar state. Figure 7c presents the simulation results of the 8 × 8 switch where the idle switches are set to the blocking state. According to the results, the worst output crosstalk for the example 8 × 8 switch is drastically suppressed by more than 50 dB, from −59.8 dB for the case of without blocking the idle switches, as shown in Fig. 7b, down to −114.4 dB for the case of blocking the idle switches, as shown in Fig. 7c. Note that in the simulations of the 8 × 8 switch, waveguide crossings are assumed to be crosstalk-free in order to isolate the impacts of the BNMZI switch on the performance of the 8 × 8 switch. This is possible in practice since the switch fabric can be built using a multi-layer SiN-on-SOI photonics platform^16^, where waveguides can cross each other on different layers and the layers can be designed to be far enough apart to eliminate crosstalk. The insertion loss for multiple inter-layer transitions across the 8 × 8 switch can be less than 1 dB (<0.07 dB per transition^16^).

## Conclusion

In this work, we have designed a high-performance photonic tri-state switch based on a BNMZI structure. The switch has a balanced architecture, in which the optical path lengths are equal in its two main interference arms, so that broadband switching can be achieved. High-speed switching can be achieved using carrier injection phase tuning. The insertion loss of carrier injection phase tuning is balanced in the BNMZI structure due to the balanced phase tuning to the switch, and hence the switch can be crosstalk-free, which, however, cannot be achieved by regular carrier injection MZI switches^1–6^. As compared with regular carrier injection MZI switches, the proposed BNMZI switch not only exhibits better performance but also provides a unique blocking functionality. An 8 × 8 dilated Banyan switch has been presented as an example to demonstrate the advantages of the blocking state in crosstalk suppression for a fully loaded *N* × *N* switch fabric, which shows dramatic suppression of more than 50 dB to the worst output crosstalk. The results of this work offer an additional degree of freedom for the manipulation of photonic switches, and may have a significant impact on the routing algorithm of *N* × *N* switches. While here our theoretical study on the BNMZI switch are targeted for high-speed applications where carrier injection phase shifters are used in the switch design, the tri-state operation also holds true for low-speed applications where thermo-optic phase shifters^17,18^ can be used in the switch design.

## Methods

The output transmissions at the cross, bar, and blocking states of the BNMZI switch, which are shown in Figs 2, 3, and 4, respectively, were calculated based on Eqs (6) and (7) using Matlab^19^. The performance of the MZI switch presented in Fig. 5a and b was calculated using Matlab based on the following transfer matrix method:

11 $$[\begin{array}{c}{E}_{Through}\\ {E}_{Cross}\end{array}]=[\begin{array}{cc}t & -j\kappa \\ -j\kappa  & t\end{array}]\,[\begin{array}{cc}{\alpha }_{1}{e}^{-j{\varphi }_{1}} & 0\\ 0 & {\alpha }_{2}{e}^{-j{\varphi }_{2}}\end{array}]\,[\begin{array}{cc}t & -j\kappa \\ -j\kappa  & t\end{array}]\,[\begin{array}{c}1\\ 0\end{array}],$$

where *E*_*Through*_ and *E*_*Cross*_ are the electric fields at the through-port and cross-port of the MZI switch, respectively. In our calculations, the 2 × 2 couplers in the MZI switch are are assumed to be lossless, i.e., *t* ^2^ + *κ* ^2^ = 1. At the cross state, no phase tuning is applied, i.e., *ϕ*_1_ = *ϕ*_2_ = 0, so we have *α*_1_ = *α*_2_ = 1. Accordingly, the through-port and cross-port transmissions are given by:

12 $$|{E}_{Through}{|}^{2}=|{t}^{2}-{\kappa }^{2}{|}^{2},$$

13 $$|{E}_{Cross}{|}^{2}=|-2j\kappa t{|}^{2}\mathrm{.}$$

At the bar state, one of the phase shifters is tuned by a *π* phase shift while the other has no phase tuning, i.e., *ϕ*_1_ = *π* and *ϕ*_2_ = 0, so we have *α*_1_ = *α* and *α*_2_ = 1. Accordingly, the through-port and cross-port transmissions are given by:

14 $$|{E}_{Through}{|}^{2}=|-\alpha {t}^{2}-{\kappa }^{2}{|}^{2},$$

15 $$|{E}_{Cross}{|}^{2}=|j\kappa t(\alpha -{\mathrm{1)|}}^{2},$$

where *α* = 0.8613 is used in the calculations.

The optical transmission simulations of the 8 × 8 dilated Banyan switch, as shown in Fig. 7, were performed using a commercially available circuit simulator, Lumerical INTERCONNECT^20^.

## Electronic supplementary material

Supplementary info
