# Supplementary material for: High-performance silicon photonic tri-state switch based on balanced nested Mach-Zehnder interferometer
Source: Sci Rep. 2017 Sep 25;7:12244. doi: 10.1038/s41598-017-12455-8 (PMC5612950; doi:10.1038/s41598-017-12455-8)
Supplement: Supplementary file 1 — Supplementary info [file 41598_2017_12455_MOESM1_ESM.pdf]

Supplementary information

High-performance silicon photonic tri-state switch based on balanced nested Mach-Zehnder interferometer

Zeqin Lu[1*], Dritan Celo[2], Hamid Mehrvar[2], Eric Bernier[2], and Lukas Chrostowski[1]

[1]Department of Electrical and Computer Engineering, University of British Columbia (UBC), Vancouver, Canada

[2]Central Research Institute, Huawei Technologies Canada Co., Ltd., Ottawa, Canada

Monte Carlo (MC) simulations can be used to analyze the BNMZI switch and MZI switch when each 2×2 coupler in the switch has a random coupling strength due to random fabrication errors. We performed MC simulations using a commercial photonics circuit simulator, Lumerical INTERCONNECT [20]. In our MC simulations, component models have been built so that each 2×2 coupler is lossless and has a random cross-coupling ratio, κ^2, between 0.48 and 0.52; each phase shifter operates as a 250 µm long carrier injection phase shifter. Supplementary Figs. 1(a), 1(b), and 1(c) show the cross state, bar state, and blocking state performance of the BNMZI switch, respectively, in 200 MC simulation trials; Supplementary Figs. 1(d) and 1(e) present the cross state and bar state performance of the MZI switch, respectively, in 200 MC simulation trials. By comparing these results, we can find that both switches have variations in their switching crosstalk due to the random κ^2 of each 2×2 coupler. At the cross state, both switches have similar switching crosstalk, as shown in Supplementary Figs. 1(a) and 1(d); at the bar state, the worst crosstalk of the BNMZI switch is almost 10 dB lower than that of the MZI switch, which are shown in Supplementary Figs. 1(b) and 1(e). Overall, the proposed BNMZI switch exhibits better performance than the MZI switch.

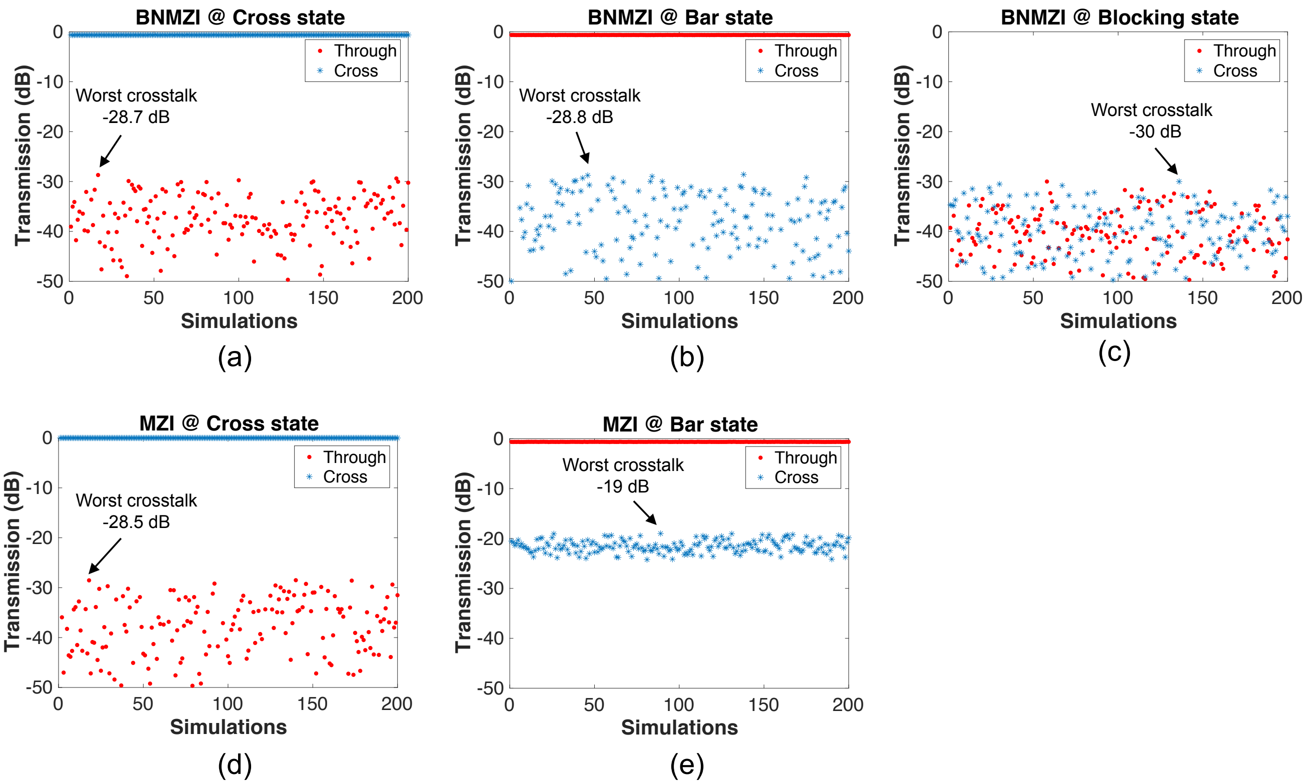

Supplementary Figure 1. (a), (b) and (c) present Monte Carlo (MC) simulation results for the BNMZI switch operating at the cross state, bar state and blocking state, respectively. (d) and (e) present MC simulation results for the MZI switch operating at the cross state and bar state, respectively.
